# Hysteresis-Free, High-Performance Polymer-Dielectric Organic Field-Effect Transistors Enabled by Supercritical Fluid

**DOI:** 10.34133/2020/6587102

**Published:** 2020-08-30

**Authors:** Yuhao Shi, Yingkai Zheng, Jialiang Wang, Ran Zhao, Tao Wang, Changbin Zhao, Kuan-Chang Chang, Hong Meng, Xinwei Wang

**Affiliations:** ^1^School of Advanced Materials, Shenzhen Graduate School, Peking University, Shenzhen 518055, China; ^2^School of Electronic and Computer Engineering, Shenzhen Graduate School, Peking University, Shenzhen 518055, China

## Abstract

Organic field-effect transistors (OFETs) are of the core units in organic electronic circuits, and the performance of OFETs replies critically on the properties of their dielectric layers. Owing to the intrinsic flexibility and natural compatibility with other organic components, organic polymers, such as poly(vinyl alcohol) (PVA), have emerged as highly interesting dielectric materials for OFETs. However, unsatisfactory issues, such as hysteresis, high subthreshold swing, and low effective carrier mobility, still considerably limit the practical applications of the polymer-dielectric OFETs for high-speed, low-voltage flexible organic circuits. This work develops a new approach of using supercritical CO_2_ fluid (SCCO_2_) treatment on PVA dielectrics to achieve remarkably high-performance polymer-dielectric OFETs. The SCCO_2_ treatment is able to completely eliminate the hysteresis in the transfer characteristics of OFETs, and it can also significantly reduce the device subthreshold slope to 0.25 V/dec and enhance the saturation regime carrier mobility to 30.2 cm^2^ V^−1^ s^−1^, of which both the numbers are remarkable for flexible polymer-dielectric OFETs. It is further demonstrated that, coupling with an organic light-emitting diode (OLED), the SCCO_2_-treated OFET is able to function very well under fast switching speed, which indicates that an excellent switching behavior of polymer-dielectric OFETs can be enabled by this SCCO_2_ approach. Considering the broad and essential applications of OFETs, we envision that this SCCO_2_ technology will have a very broad spectrum of applications for organic electronics, especially for high refresh rate and low-voltage flexible display devices.

## 1. Introduction

Organic electronic devices have recently aroused great attention for their high mechanical flexibility and low fabrication cost with diverse important functionalities [1–4]. In organic circuits, organic field-effect transistors (OFETs) are of the core units, which are essential for a huge number of practical applications, such as flexible displays [5, 6], radiofrequency identification tags [7, 8], wearable sensors [9, 10], and biocompatible devices [11, 12]. The performance of the OFET devices replies critically on the properties of the dielectric layers in them. For instance, the polarization, purity, and charge traps of the dielectrics strongly affect the hysteresis, subthreshold swing, leakage and on/off currents, and effective carrier mobility of OFETs, thereby affecting the switching speed, power consumption, and stability of the organic circuits [13–16]. Moreover, the mechanical properties of the dielectrics are also very important, especially for flexible applications such as rollable displays [17, 18]. The materials of the dielectrics for OFETs are often inorganic oxides, organic polymers, and their mixtures or multilayers [13]. Among these materials, the organic polymer dielectrics are of particular interest, because they are intrinsically flexible and also naturally compatible with other organic components in devices [14, 19]. These merits are particularly important for flexible applications, such as rollable displays, where bending the devices down to <1 mm in radius is required and the inorganic dielectrics are difficult to meet this requirement [18, 20–22].

As a representative polymer dielectric, poly(vinyl alcohol) (PVA) is of particularly common interest for the use in OFETs [22–25]. PVA has a simple structure and of low manufacturing cost, and it also has a relatively high dielectric constant and can be easily spin-coated to afford films. However, PVA contains moisture-sensitive hydroxyl groups, which can act as electron traps causing issues of hysteresis, leakage, and stability for OFETs [26, 27], and also sodium impurities (hundreds ppm level) are almost inevitable in PVA because NaOH is used in the synthesis of PVA from hydrolyzing poly(vinyl acetate) [15, 28, 29]. Previous works showed that the dielectric performance of PVA could be improved by UV irradiation or annealing in air with a cross-linking agent such as ammonium dichromate or glutaraldehyde [22–24, 30], but this approach is not very effective in removing the hydroxyls and apparently it cannot remove the ionic impurities in PVA. Consequently, the device performance such as hysteresis, subthreshold swing, and effective carrier mobility is still not satisfactory, which considerably limits the practical applications of the polymer-dielectric OFETs for high-speed, low-voltage flexible organic circuits.

In this work, we develop a new approach of using a supercritical CO_2_ fluid (SCCO_2_) to treat PVA to boost its dielectric performance. Supercritical fluids have combined properties of vapor and liquid; their densities and viscosities are lower than liquids and their diffusivities are high like gases, so the transfer of mass in supercritical fluids is very rapid [31–33]. With an additive of desiccant, such as anhydrous calcium chloride, the supercritical CO_2_ fluid can remove water and other impurities in a fast and efficient way [34–36]. As demonstrated in the following, benefited from the merits of the supercritical fluids, the dielectric performance of PVA is significantly boosted by the SCCO_2_ treatment. The treatment is able to completely eliminate the hysteresis of the PVA OFETs and also significantly reduce the device subthreshold slope to a very low number of 0.25 V/dec while boosting the saturation regime carrier mobility to a very high number of 30.2 cm^2^ V^−1^ s^−1^. Both of these numbers are quite remarkable for flexible OFETs with polymer dielectrics. We further demonstrate that, coupling with an organic light-emitting diode (OLED), the OFET using the SCCO_2_-treated PVA dielectric is able to function very well under fast switching speed, which suggests that this SCCO_2_ approach is of high promise for future applications in organic electronics, especially for high refresh rate flexible display devices.

## 2. Results

**Figure 1:**
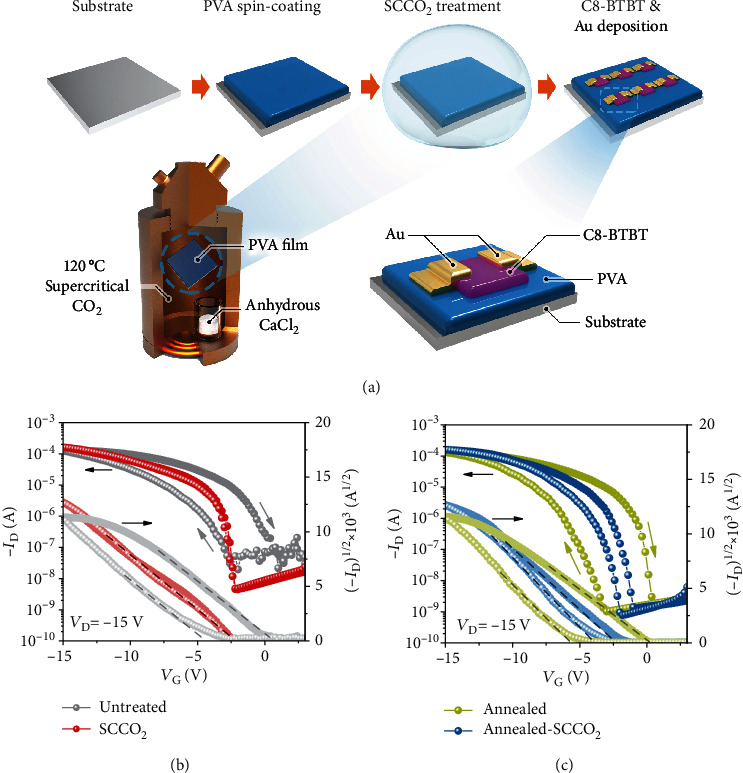
(a) Schematic illustration of the OFET device fabrication process, where the PVA dielectric is treated with supercritical CO_2_ fluid. (b) Representative transfer characteristics of the OFET devices with untreated and SCCO_2_-treated PVA dielectrics. (c) Representative transfer characteristics of the OFET devices with air-annealed and sequentially air-annealed and SCCO_2_-treated PVA dielectrics.

Standard bottom-gate/top-contact OFET devices were used to prove the concept in this work. The devices used 2,7-dioctyl[1]benzothieno[3,2-b][1]benzothiophene (C8-BTBT) as the representative organic semiconductor and PVA as the polymer dielectric. The fabrication process of the devices is schematically illustrated in Figure 1(a), where a ~340 nm PVA layer was first spin-coated on a Si substrate, and then, the layer was treated with supercritical CO_2_ fluid (i.e., SCCO_2_ treatment) at 120°C under 3000 psi for 1 hour with the additive of anhydrous calcium chloride as the desiccant. The OFET devices were then completed by sequential evaporation of the C8-BTBT semiconductor and Au source/drain metal contacts. For comparison purposes, we also fabricated the OFET devices with alternative dielectrics of the untreated (as spin-coated) PVA, air-annealed PVA (at 120°C for 1 h), and sequentially air-annealed and SCCO_2_-treated PVA.

The electrical performance of the fabricated OFET devices was carefully characterized, and representative transfer characteristics in the saturated region are compared in Figures 1(b) and 1(c). The transfer characteristics were measured by sweeping the gate voltage (*V*_G_) from +3 V (off-state) to −15 V (on-state) and then back to +3 V, while keeping fixed the drain voltage (*V*_D_) at −15 V for generating saturated drain current (*I*_D_) (Figure S1). As shown in Figure 1(b), pronounced hysteresis in the *I*_D_‐*V*_G_ curve appeared for the devices using the untreated PVA dielectric, whereas the hysteresis was greatly remedied by the SCCO_2_ treatment on the PVA dielectric. To quantify the hysteresis, we extracted the threshold voltages (*V*_th_) for both the forward and backward *V*_G_ sweeps and took their difference (Δ*V*_th_) as a measure of the hysteresis. With the statistical analysis over 20 devices, we found that Δ*V*_th_ was fairly large as 5.55 ± 2.60 V for the untreated PVA devices and it became virtually zero (0.00 ± 0.02 V) by the addition of the SCCO_2_ treatment. In contrast, conventional air annealing of the PVA dielectric did not improve the hysteresis at all, as Δ*V*_th_ remained at 5.72 ± 0.50 V for the air-annealed devices (Figure 1(c)). Notably, we also found that if the same SCCO_2_ treatment was carried out after the air-annealing step, the hysteresis could also be improved to some extent (Δ*V*_th_ reduced to 1.52 ± 0.34 V) but not as much as that without the air-annealing step (Figure 1(c)). These numbers along with other device characteristics are summarized in Table S1.

The SCCO_2_ treatment was also found to significantly improve the subthreshold slope of the devices. The subthreshold slope (SS) is defined as SS = *dV*_G_/*d*(log_10_*I*_D_), which reflects the increase of *V*_G_ needed to generate one order of magnitude increase in the drain current. Certainly, a low SS is favored for the device operation with low voltage, high switching speed, and low power consumption. As shown in Figure 1(b) and Table S1, the devices with the untreated PVA dielectric exhibited a fairly large SS of 2.25 ± 0.51 V/dec. By adopting the SCCO_2_ treatment, the SS value was significantly improved to 0.43 ± 0.21 V/dec, which is a remarkably low number for polymer-dielectric OFETs. In contrast, conventional air annealing could only reduce the SS value to some extent, and the residual SS was still high (1.29 ± 0.41 V/dec). If the SCCO_2_ treatment was conducted after the air annealing, not much further improvement in SS could be obtained (0.93 ± 0.25 V/dec). The SCCO_2_ treatment could also improve both on- and off-state drain currents (*I*_on_ and *I*_off_). Compared to the devices using the untreated PVA, the SCCO_2_-treated devices showed an enhanced *I*_on_ from 126 ± 14 to 160 ± 8 *μ*A (at *V*_G_ = −15 V) and a suppressed *I*_off_ from 2.0 × 10^−8^ to 4.8 × 10^−9^ A, and therefore, the *I*_on_/*I*_off_ ratio was improved by approximately 5 times from 6.3 × 10^3^ to 3.3 × 10^4^. It is worth noting that although *I*_off_ could also be improved by air annealing, the promotion of *I*_on_ could only be achieved by the SCCO_2_ treatment. In addition, the saturation regime carrier mobility (*μ*_sat_) of the devices was also found to increase from 20.9 ± 4.8 to 22.1 ± 2.9 cm^2^ V^−1^ s^−1^ by the SCCO_2_ treatment, whereas the conventional air annealing did not appear to improve *μ*_sat_ (Table S1). It is worth noting that the extraction of the carrier mobility from the saturation regime (*μ*_sat_) could possibly overestimate the numbers for high-mobility OFETs [37]; Choi et al. [38] recommended to look into the measurement reliability factor (*r*, see Methods) and calculate the corresponding effective carrier mobility (*μ*_eff_), defined as *r* × *μ*_sat_. Therefore, we also calculated the effective mobility for each OFET, and the number was found to also increase by the SCCO_2_ treatment from 9.7 ± 3.2 to 14.0 ± 1.8 cm^2^ V^−1^ s^−1^, as compared to the untreated devices (Table S1). The bias stress stability of the OFETs was also tested, and the SCCO_2_-treated devices exhibited much improved stability as compared to the untreated devices (Figure S2). To sum up the device characteristic results, we found that the SCCO_2_ treatment could considerably improve the dielectric properties of PVA and therefore significantly enhance the device performance of OFETs. With the use of the SCCO_2_-treated PVA dielectric, the OFET devices showed virtually zero hysteresis in *I*_D_‐*V*_G_ and a remarkably low SS of 0.43 V/dec, along with other appreciably improved parameters of *I*_on_, *I*_on_/*I*_off_ ratio, and *μ*_sat_.

**Figure 2:**
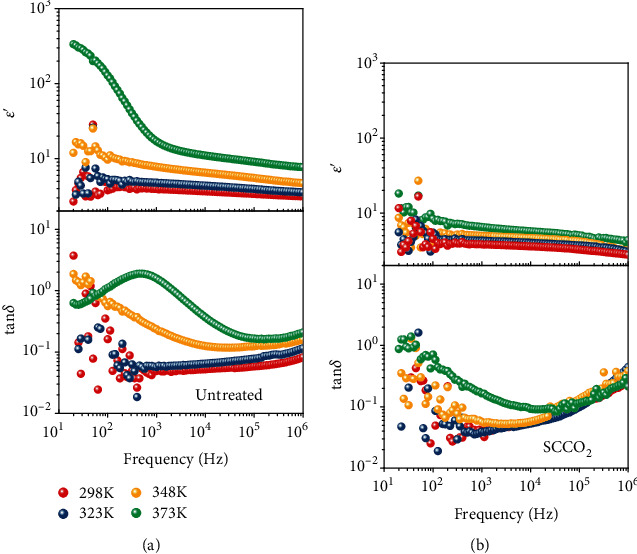
Dielectric spectra of (a) the untreated and (b) SCCO_2_-treated PVA layers measured at various temperatures from 298 to 373 K.

To further understand the associated mechanisms, we looked again at the direction of the *I*_D_‐*V*_G_ hysteresis for the untreated PVA devices. As shown in Figure 1(b), the hysteresis loop was in the clockwise direction, i.e., the current in the backward voltage sweep was higher than that in the forward sweep. Given this direction, the hysteresis was possibly originated from three sources [16], which are the polarization of the dielectric (ferroelectric or quasi-ferroelectric), charge injection from the gate electrode, and/or presence of mobile ions in the dielectric. The polarization of the dielectric was unlikely the reason because PVA is not a ferroelectric material [39]. The effect from the charge injection from the gate is usually prominent for thin dielectric layers (on the order of nanometers) [40] but unlikely to be significant for thick dielectrics as used in this case. Therefore, the presence of mobile ions in the dielectric seems to be the most likely dominating source for the hysteresis, and indeed, it has been used to explain the hysteresis in several other works [15, 16, 40]. To verify this mechanism, we fabricated metal-insulator-semiconductor (MIS) capacitor devices using the corresponding untreated and SCCO_2_-treated PVA as the dielectric layers and measured their dielectric spectra at various temperatures from 298 to 373 K. As displayed in Figure 2, the real part of the obtained dielectric permittivity (*ε*′) and the loss tangent (tan *δ*) of the PVA dielectrics are plotted with respect to the frequency of the ac excitation voltage from 20 to 10^6^ Hz. For the untreated PVA dielectric (Figure 2(a)), the *ε*′ curve at 373 K is particularly large in the low-frequency region (<10^2^ Hz), which signifies an additional relaxation channel for a slow responsive process [41, 42]. The tan *δ* curve at 373 K also shows a typical loss peak at 10^3^ Hz for this process [41, 42]. The relaxation time of this slow process is consistent with the movement of mobile ions in a polymer matrix [40, 41], and the power law exponent of the imaginary part of the dielectric permittivity (*ε*^″^) further suggests that the ion movement was mixed of drifting and hopping (Figure S3). At lower temperatures (298~323 K), the ion movement became even slower, so the characteristic relaxation time was beyond the frequency range measured herein; nevertheless, the tan *δ* curve at 323 K shows some tail-like feature of a loss peak. As for the SCCO_2_-treated dielectric (Figure 2(b)), the change of *ε*′ with temperature and frequency is much smaller, and the tan *δ* curves show no loss peaks, which are consistent with the removal of the mobile ions by the SCCO_2_ treatment. The mobile ions in polymers are usually monovalent ions, such as Na^+^, K^+^, and Li^+^. In PVA, it is likely the Na^+^ ions that cause the hysteresis issue, because NaOH is actually used in the synthesis of PVA from hydrolyzing poly(vinyl acetate), and therefore, it is very difficult to completely remove the Na^+^ ions in the final product. The Na^+^ ions remained in the PVA dielectric layer, causing the hysteresis in the OFET *I*_D_‐*V*_G_ response; as the SCCO_2_ treatment removed the Na^+^ ions, the hysteresis problem was therefore remedied.

**Figure 3:**
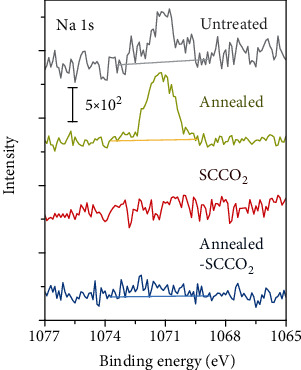
XPS Na 1s spectra of the untreated, SCCO_2_-treated, air-annealed, and sequentially air-annealed and SCCO_2_-treated PVA films.

The presence and removal of the Na^+^ ions in the PVA layer were further confirmed by X-ray photoelectron spectroscopy (XPS) and by X-ray fluorescence spectrometry (XRF).  Figure 3 displays the high-resolution XPS spectra of the Na 1s core-level emission for an as-spin-coated PVA film and the films subjected to each treatment as aforementioned. As shown in Figure 3, both the untreated and air-annealed PVA films exhibited a prominent Na 1s peak, and the peak intensity was greatly reduced by the SCCO_2_ treatment. In particular, the Na XPS signal was below the noise level for the PVA film subject to only the SCCO_2_ treatment (detection limit ~0.01 at.%). XRF was further used to quantify the Na contents inside the films. XRF measures the overall Na content throughout a film and is comparatively more accurate in quantifying metal elements. The Na contents were measured to be 141 ± 24, 172 ± 27, 4 ± 20, and 42 ± 21 ppm in mass for the untreated, air-annealed, SCCO_2_-treated, and sequentially air-annealed and SCCO_2_-treated PVA, respectively. These results clearly indicate that the SCCO_2_ treatment was very effective in removing the Na^+^ ions in the PVA film, as the Na content after the SCCO_2_ treatment was barely observable by XRF. We also noticed that if the PVA film was first annealed, the thereafter SCCO_2_ treatment became not as effective as before, possibly because the air annealing densified the PVA film and made some Na^+^ ions locally trapped and inaccessible by the supercritical fluid. Overall, the Na content was in positive correlation with the magnitude of the hysteresis (Δ*V*_th_) as shown Figure 1. Therefore, these results well corroborate that the residual Na^+^ ions in the PVA layer were the major origin for the *I*_D_‐*V*_G_ hysteresis and those ions could be well removed by the SCCO_2_ treatment.

**Figure 4:**
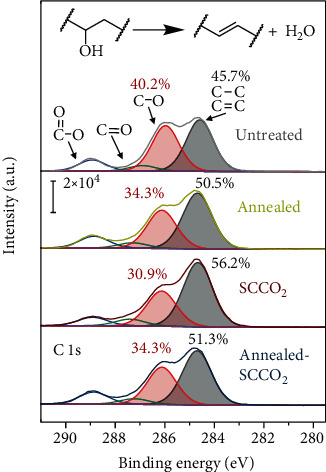
XPS C 1s spectra of the untreated, SCCO_2_-treated, air-annealed, and sequentially air-annealed and SCCO_2_-treated PVA films.

As for the subthreshold slope, it is known that SS is affected by the density of the charge traps at the semiconductor-insulator interface (*D*_it_) and in the bulk of the semiconductor (*D*_bulk_) via $\text{SS}=\ln\left(10\right)\left(kT/q\right)\left(1+\left(\left(q\sqrt{\varepsilon_{\text{SC}}D_{\text{bulk}}}+q^{2}D_{\text{it}}\right)/C_{\text{i}}\right)\right)$, where *k*, *T*, *q*, and *C*_i_ are, respectively, the Boltzmann constant, temperature, elementary charge constant, and the areal capacitance of the gate dielectric [43]. Provided that *C*_i_ was roughly the same before and after the SCCO_2_ treatment (Table S2), it is likely that the charge trap density (*D*_it_ and/or *D*_bulk_) was greatly reduced by the SCCO_2_ treatment. The charge traps in PVA are probably from its hydroxyl groups, as they have been found to be efficient in trapping electrons and affect the device performance [26, 27]. To this end, we conducted XPS measurements to investigate the change of PVA upon the above treatments.  Figure 4 displays the results of the XPS C 1s spectra for the untreated, air-annealed, SCCO_2_-treated, and sequentially air-annealed and SCCO_2_-treated PVA samples. All the spectra could be deconvoluted into four peak components at approximately 284.6, 286.0, 287.3, and 288.9 eV in binding energy, and these peak components could be assigned to the carbons in C−C/C=C, C−O, C=O, and O−C=O moieties, respectively [44]. As compared in Figure 4, both the air-annealing and SCCO_2_ treatments reduced the C−O intensity and increased the C−C/C=C intensity, while the C=O and O−C=O intensities remained roughly unchanged. This observation is suggestive of a dehydration process, which removes the hydroxyl groups and converts C−O to C=C as schematically illustrated in Figure 4. The relative intensities of the C−O and C−C/C=C peak components are denoted above the peaks, and comparatively, the SCCO_2_ treatment appears to be the most effective toward the dehydration, as the corresponding C−O and C−C/C=C intensities are, respectively, the lowest and highest among the others. The removal of the hydroxyl groups was also corroborated by Fourier-transform infrared spectroscopy (Figure S4), where the absorbance of the hydroxyl stretching mode was significantly reduced after the above treatments and the SCCO_2_ treatment gave out the lowest hydroxyl absorbance. Although the detailed mechanisms for the SCCO_2_ treatment being most efficient toward the dehydration are unknown and probably complex, we consider that the enhanced mass transfer of water by supercritical CO_2_ fluid was likely to boost the dehydration reaction according to Le Chatelier's principle [31]. It is worth noting that the SCCO_2_ treatment became ineffective post the air annealing, and this is perhaps because at the same time of dehydration the PVA chains also cross-linked and created some small isolated zones that trapped water from being further removed [45]. Nevertheless, the SCCO_2_ treatment was the most effective in removing the electrically detrimental hydroxyls in PVA, which therefore considerably reduced the charge trap density and improved the subthreshold slope of the devices.

**Figure 5:**
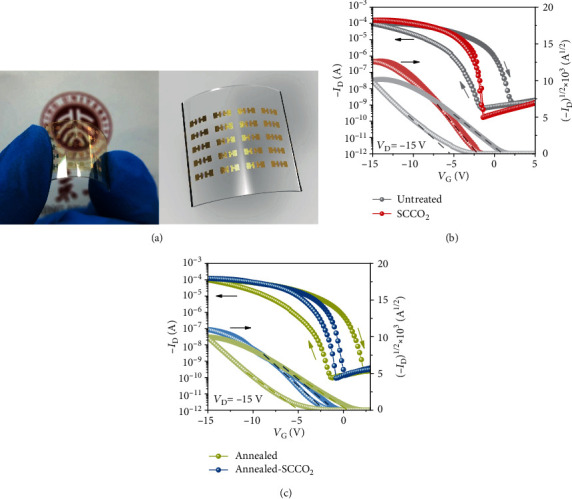
(a) Photographs of the OFETs prepared on a flexible transparent ITO-coated plastic substrate. (b) Transfer characteristics of the flexible OFETs with untreated and SCCO_2_-treated PVA dielectrics. (c) Transfer characteristics of the flexible OFETs with air-annealed and sequentially air-annealed and SCCO_2_-treated PVA dielectrics.

In the following, we further demonstrate that the SCCO_2_ approach is also well applicable to flexible OFET devices, which is of high technological importance for future applications. Although polymer dielectrics are ideal for flexible organic electronics, the problematic hysteresis associated with the polymer dielectrics has long been a critical issue that limits the switching speed, operation voltage, and power consumption of the OFET devices [13–16, 46, 47]. We herein used a piece of flexible transparent plastic coated with indium tin oxide (ITO) as the substrate and back gate and fabricated the same OFET devices on it, as shown in Figure 5(a). During the device fabrication, the same treatments of air annealing, SCCO_2_ treatment, and sequentially air annealing and SCCO_2_ treatment were carried out on the PVA dielectrics, and the associated device performances (transfer characteristics) are shown in Figures 5(b) and 5(c). Similar to those shown in Figure 1, significant improvement in the device performance was also observed for the flexible OFETs: benefited from the SCCO_2_ treatment, the hysteresis in the *I*_D_‐*V*_G_ curve was essentially removed (Δ*V*_th_ = 0.03 ± 0.07 V), the subthreshold slope was largely reduced to 0.25 ± 0.11 V/dec, *I*_on_ was enhanced to 158 ± 28 *μ*A, *I*_off_ was reduced to 1.7 × 10^−10^ A, and therefore, the *I*_on_/*I*_off_ ratio was significantly improved to 9.2 × 10^5^ (Table S3). Notably, the SS and *I*_on_/*I*_off_ numbers were even better than those on Si substrates, which is perhaps because the oxygen-rich ITO could also partially oxidize the hydroxyls and therefore reduced the charge trap density and suppressed the leakage pathway [48]. As a result, a very high saturation regime mobility (*μ*_sat_) of 30.2 ± 4.6 cm^2^ V^−1^ s^−1^ and a high effective mobility (*μ*_eff_) of 13.8 ± 2.1 cm^2^ V^−1^ s^−1^ were obtained, and these numbers considerably outperformed all previously reported flexible OFET devices, except for a recent work [49] which used single-crystal C8-BTBT as the organic semiconductor and reported a comparable carrier mobility (33.4 and 13.3 cm^2^ V^−1^ s^−1^ for *μ*_sat_ and *μ*_eff_, respectively) (Table S4). Given that the single crystal is considered to be the intrinsic limit for enhancing the mobility, our outcome relying on the improvement of the dielectric layer is quite remarkable in approaching to this limit.

**Figure 6:**
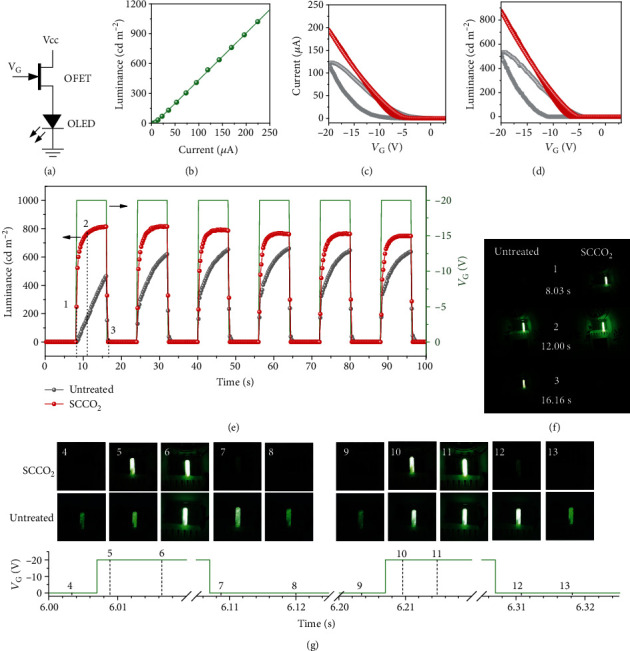
(a) Circuit diagram showing an OFET in series with an OLED. (b) Luminance of the OLED as the function of the drive current. (c) Current and (d) luminance as the functions of the gate voltage applied on the OFETs (*V*_CC_ = −30 V). The OFETs were fabricated with untreated and SCCO_2_-treated PVA dielectrics. (e) Traces of luminance with time and (f) representative snapshots showing the switching behaviors of the OFETs upon square-wave *V*_G_ of 16 s in period. (g) Snapshots of the OLED upon faster switching *V*_G_ of 0.2 s in period (i.e., 5 Hz square wave). The corresponding time points are illustrated in the bottom curve.

As a further demonstration of the SCCO_2_-prepared OFET devices for practical applications in organic electronics, we built a circuit using a SCCO_2_-prepared OFET in series with a green-color OLED, where the OFET served as a switch to control the OLED (Figure 6(a)). As for demonstrative purposes, the green-color OLED was fabricated of a fairly large area of 16 mm^2^, which is much larger than usual pixel size used in displays [50]; and therefore, as shown in Figure 6(b), the OLED needed a fairly large drive current of around 100 *μ*A to achieve a luminance of 500 cd m^−2^ as needed for typical cell phone displays [51]. The circuit operation results are shown in Figures 6(c) and 6(d), where the current and luminance of the OLED are plotted against the gate voltage applied on the OFET, and the gate voltage was swept from 3 to −20 V and then back to 3 V. For comparison purposes, an OFET prepared with the untreated PVA dielectric was also used for these experiments, and the results are also plotted in Figures 6(c) and 6(d) for benchmarking. Clearly, significantly higher current and luminance along with much improved hysteresis were obtained for the OLED with the SCCO_2_-treated OFET. Next, we supplied square-wave voltage alternating at 0 and −20 V (16 s in period) on the OFET gate to examine its switching behavior.  Figure 6(e) compares the traces of the OLED luminance, and the associated video is provided as Supplementary Video 1. Clearly, the OLED coupled with the SCCO_2_-treated OFET exhibited better performance in both the luminance and switching speed. A closer comparison of the snapshots taken at 0.03 and 0.16 s after switching *V*_G_ (Figure 6(f)) clearly shows that the untreated OFET resulted in apparent delay of light emitting when turning on and presence of ghost image when turning off, but these problems were not present for the SCCO_2_-treated OFET. Another set of experiments was carried out using a much faster alternation speed for *V*_G_, i.e., 5 Hz square wave (0.2 s in period). The associated video is provided as Supplementary Video 2, and a number of the snapshots taken at various times are comparatively shown in Figure 6(g). Apparently, the untreated OFET was not able to follow the fast switching of *V*_G_ as the ghost image persisted throughout the entire off duration; and this problem was well conquered by the SCCO_2_ treatment as no such ghost image appeared for the SCCO_2_-treated device. All these above results clearly demonstrated that the SCCO_2_ treatment was very effective to significantly improve the switching behavior of the OFETs for display control applications. Considering that OFETs are of the core units in a huge variety of contemporary organic circuits, we envision that this SCCO_2_ technology will have a very broad spectrum of applications for organic electronics, especially for high refresh rate and low-voltage flexible display devices.

## 3. Discussion

In summary, we developed a new approach of using supercritical CO_2_ fluid (SCCO_2_) treatment on PVA dielectrics to achieve remarkably high-performance polymer-dielectric OFETs. Using C8-BTBT as the representative OFET semiconductor material, we demonstrated that the SCCO_2_ treatment on PVA was able to completely eliminate the hysteresis in the OFET transfer *I*_D_‐*V*_G_ curves, which is a very critical issue for polymer-dielectric OFETs in general. Meanwhile, the treatment could also significantly reduce the OFET subthreshold slope and improve the on/off currents and carrier mobility. Detailed mechanism studies suggested that the effective elimination of the hysteresis and significant reduction of the subthreshold slope were because of the efficient removal of the mobile Na^+^ ions and hydroxyl groups by the supercritical CO_2_ fluid. We further showed that this SCCO_2_ approach was also well applicable to flexible OFETs, where not only was the *I*_D_‐*V*_G_ hysteresis successfully removed but we were also able to achieve a very low subthreshold slope of 0.25 V/dec with a very high saturation regime carrier mobility of 30.2 cm^2^ V^−1^ s^−1^, and both of these numbers are quite remarkable for the flexible OFETs with polymer dielectrics. We further demonstrated that, coupling with an OLED, the SCCO_2_-treated OFET was able to function very well under a fast switching speed of 5 Hz, which clearly indicated that an excellent switching behavior of polymer-dielectric OFETs could be enabled by this SCCO_2_ approach. Considering that OFETs are of the core units in a huge variety of contemporary organic circuits, we envision that this SCCO_2_ technology will have a very broad spectrum of applications for organic electronics, especially for high refresh rate and low-voltage flexible display devices.

## 4. Materials and Methods

### 4.1. Device Fabrication

To fabricate OFETs, heavily doped *p*-type Si wafers or ITO-coated polyethylene terephthalate (PET) plastic sheets were used as the bottom-gate substrates. The substrates were first cleaned by sequentially ultrasonicating in acetone, isopropanol, and deionized water for 20 min each and then treated with UV/ozone for 15 min. The PVA dielectric layer was prepared by spin coating a PVA solution on the substrates at a speed of 3000 rpm. The PVA solution was prepared by first dissolving PVA solid particles (Mowiol® 40-88, 205,000 in molecular weight, Sigma-Aldrich) in deionized water (7.0 wt.%) at 70°C for 4 h and then adding ammonium dichromate (10 wt.% of PVA) for cross-linking. The PVA layer was then treated with supercritical CO_2_ fluid with the additive of anhydrous calcium chloride as the desiccant at 120°C under 3000 psi for 1 h (i.e., SCCO_2_ treatment). For comparison purposes, the PVA layer was alternatively annealed in air at 120°C for 1 h or sequentially annealed in air and then subjected to the same SCCO_2_ treatment as above. In addition, the as-spin-coated PVA (i.e., untreated PVA) was also used for comparison. The organic semiconductor layer (30 nm) of 2,7-dioctyl[1]benzothieno[3,2-b][1]benzothiophene (C8-BTBT) was prepared by thermal evaporation at a deposition rate of 0.5 Å s^−1^ under chamber base pressure of 2 × 10^−6^ Torr. The OFET devices were then completed by thermal evaporation of 50 nm Au via a shadow mask to define the source and drain regions. The channel length and width of the OFETs were 100 *μ*m and 1000 *μ*m, respectively. The green-color OLEDs were fabricated following a previous report [52]. The layer structure of the OLED was ITO/HATCN (5 nm)/*N*,*N*′-bis(naphthalen-1-yl)-*N*,*N*′-bis(phenyl)-benzidine (30 nm)/*N*,*N*-bis(4-(dibenzo[b,d]furan-4-yl)phenyl)[1,1′:4′,1^″^-terphenyl]-4-amine (10 nm)/DMIC-CZ:DMIC-TRZ:Ir(ppy)_2_acac (40 nm)/ANT-BIZ (70 nm)/Liq (2.5 nm)/Al (100 nm), and the layers were sequentially deposited by thermal evaporation. MIS capacitors were also prepared on the heavily doped Si wafer with the aforementioned PVA dielectrics. The bottom Si served as the bottom electrodes, and the top electrodes were 500 *μ*m diameter circles of 50 nm thick Au, which were prepared by thermal evaporation via a shadow mask.

### 4.2. Characterizations

The electrical characterizations of the OFETs were carried out on a standard probe station with a Keysight B1500A semiconductor device analyzer in ambient air. Field-effect hole mobility in saturation regime (*μ*_sat_) and threshold voltage (*V*_th_) were extracted via the relation *I*_D_ = −(*W*/2*L*)*μ*_FE_*C*_i_(*V*_G_ − *V*_th_)^2^, where *W*, *L*, and *C*_i_ are the channel width, channel length, and areal capacitance of the gate dielectric, respectively. The effective carrier mobility (*μ*_eff_) was calculated by *μ*_eff_ = *r* × *μ*_sat_, where *r* is the reliability factor defined as [38] $r={\left[\left(\sqrt{\left|I_{\text{on}}\right|}-\sqrt{\left|I_{\text{off}}\right|}\right)/{\left|V_{\text{G}}\right|}^{\max}\right]}^{2}/\left(\left({WC}_{\text{i}}/2L\right)\mu_{\text{sat}}\right)$. The dielectric spectra were measured by a Keysight E4980A LCR meter on the MIS capacitors with series resistance corrected. The luminance of the OLEDs was measured by a TOPCON BM-7AS luminance colorimeter. To characterize the material properties of the PVA layer, we used XPS (Thermo Scientific, Escalab 250Xi), XRF (Rigaku, ZSX Primus II), and Fourier-transform infrared spectroscopy (FTIR) (PerkinElmer, Frontier), and the layer thickness was measured by a stylus profiler (KLA Tencor, D-600).

## Supplementary Materials

Supplementary Materials Figure S1: representative output characteristics of the OFET devices with (a) untreated, (b) SCCO_2_-treated, (c) air-annealed, and (d) sequentially air-annealed and SCCO_2_-treated PVA dielectrics. Figure S2: comparison of the bias stress stability of the OFET devices with (a) untreated and (b) SCCO_2_-treated PVA dielectrics. During the bias stress test, the gate voltage was biased at –20 V, and the transfer characteristic was measured for every 5 min. Table S1: summary of the device characteristics for the PVA OFETs fabricated on Si substrates. The statistics were obtained based on the analysis over 20 devices. Figure S3: imaginary part of the dielectric permittivity (*ε*^″^) of the untreated PVA (at 373 K). Table S2: thickness (*d*) and areal capacitance (*C*_i_) of the gate dielectric upon various treatments. Figure S4: FTIR spectra of the untreated, SCCO_2_-treated, air-annealed, and sequentially air-annealed and SCCO_2_-treated PVA films. Table S3: summary of the device characteristics for the PVA OFETs fabricated on flexible ITO-coated plastic substrates. Table S4: comparison of the device performance of the reported flexible OFETs using polymer dielectrics. Table S5: comparison of the device performance of the reported OFETs using the C8-BTBT semiconductor. Supplementary Video 1: switching behavior of the OFET-OLED circuit under the square-wave *V*_G_ of 16 s in period. Supplementary Video 2: switching behavior of the OFET-OLED circuit under the square-wave *V*_G_ of 0.2 s in period (i.e., 5 Hz square wave).
