# Supplementary material for: Hysteresis-Free, High-Performance Polymer-Dielectric Organic Field-Effect Transistors Enabled by Supercritical Fluid
Source: Research (Wash D C). 2020 Aug 30;2020:6587102. doi: 10.34133/2020/6587102 (PMC7510345; doi:10.34133/2020/6587102)
Supplement: Supplementary Materials — Figure S1: representative output characteristics of the OFET devices with (a) untreated, (b) SCCO2-treated, (c) air-annealed, and (d) sequentially air-annealed and SCCO2-treated PVA dielectrics. Figure S2: comparison of the bias stress stability of the OFET devices with (a) untreated and (b) SCCO2-treated PVA dielectrics. During the bias stress test, the gate voltage was biased at –20 V, and the transfer characteristic was measured for every 5 min. Table S1: summary of the device characteristics for the PVA OFETs fabricated on Si substrates. The statistics were obtained based on the analysis over 20 devices. Figure S3: imaginary part of the dielectric permittivity (ε″) of the untreated PVA (at 373 K). Table S2: thickness (d) and areal capacitance (Ci) of the gate dielectric upon various treatments. Figure S4: FTIR spectra of the untreated, SCCO2-treated, air-annealed, and sequentially air-annealed and SCCO2-treated PVA films. Table S3: summary of the device characteristics for the PVA OFETs fabricated on flexible ITO-coated plastic substrates. Table S4: comparison of the device performance of the reported flexible OFETs using polymer dielectrics. Table S5: comparison of the device performance of the reported OFETs using the C8-BTBT semiconductor. Supplementary Video 1: switching behavior of the OFET-OLED circuit under the square-wave VG of 16 s in period. Supplementary Video 2: switching behavior of the OFET-OLED circuit under the square-wave VG of 0.2 s in period (i.e., 5 Hz square wave). [file 6587102.f1.zip › SI 20200807.pdf]

Supplementary materials

Hysteresis-Free, High-Performance Polymer-Dielectric Organic Field-Effect Transistors Enabled by Supercritical Fluid

Yuhao Shi[1], Yingkai Zheng[2], Jialiang Wang[1], Ran Zhao[1], Tao Wang[1], Changbin Zhao[1], Kuan-Chang Chang[2], Hong Meng[1] and Xinwei Wang[1]**

[1]School of Advanced Materials, Shenzhen Graduate School, Peking University, Shenzhen 518055, China
[2]School of Electronic and Computer Engineering, Shenzhen Graduate School, Peking University, Shenzhen 518055, China
*Correspondence should be addressed to Hong Meng; menghong@pku.edu.cn and Xinwei Wang; wangxw@pkusz.edu.cn

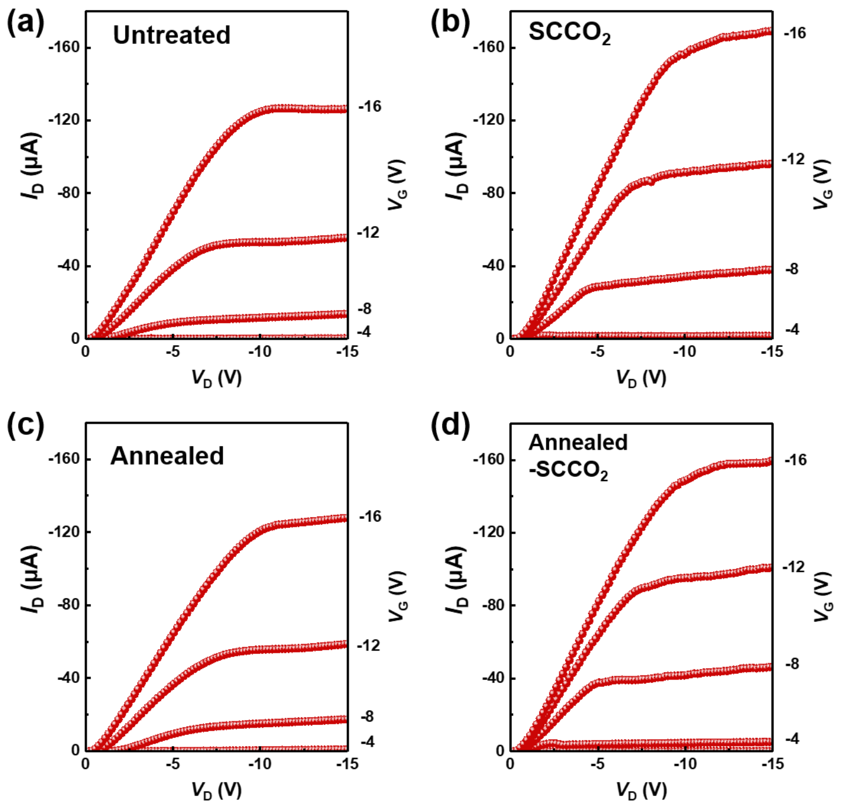

Figure S1. Representative output characteristics of the OFET devices with (a) untreated, (b) $SCCO_2$-treated, (c) air-annealed, and (d) sequentially air-annealed and $SCCO_2$-treated PVA dielectrics.

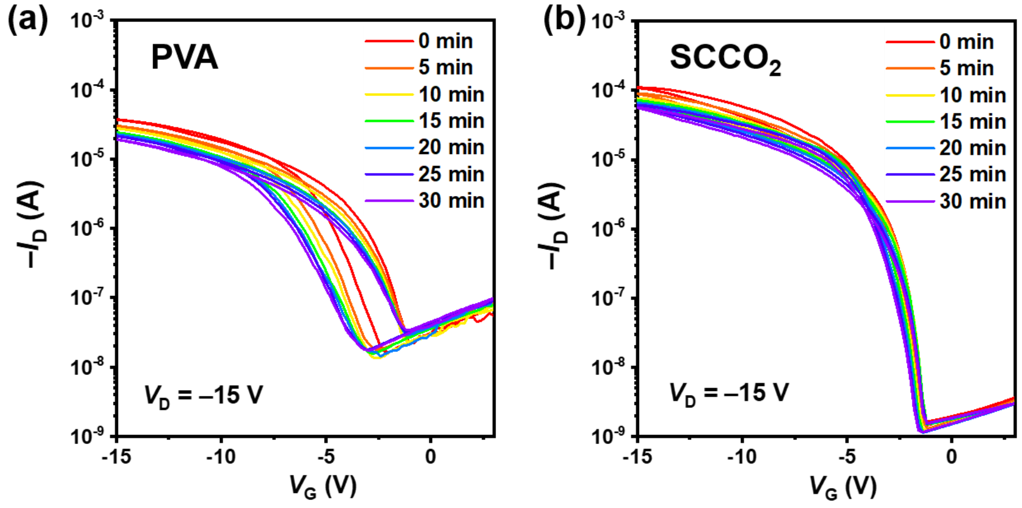

Figure S2. Comparison of the bias stress stability of the OFET devices with (a) untreated and (b) $SCCO_2$-treated PVA dielectrics. During the bias stress test, the gate voltage was biased at –20 V, and the transfer characteristic was measured for every 5 min.

Table S1. Summary of the device characteristics for the PVA OFETs fabricated on Si substrates. The statistics were obtained based on the analysis over 20 devices.

	V_{th} forward (V)	ΔV_{th}	$SS_{forward}$ (V/dec)	I_{on} (nA μm^{-1})	I_{on}/I_{off}	μ_{sat} (cm^2 V^{-1} s^{-1})	μ_{eff} (cm^2 V^{-1} s^{-1})
Untreated	−4.71	5.55±2.60	2.25±0.51	126±14	6.3×10^3	20.9±4.8	9.7±3.2
$SCCO_2$	−2.40	0.00±0.02	0.43±0.21	160±8	3.3×10^4	22.1±2.9	14.0±1.8
Annealed	−5.43	5.72±0.50	1.29±0.41	132±5	1.3×10^5	20.5±2.1	10.8±1.1
Annealed -$SCCO_2$	−3.75	1.52±0.34	0.93±0.25	160±3	2.1×10^5	21.0±2.0	13.3±1.3

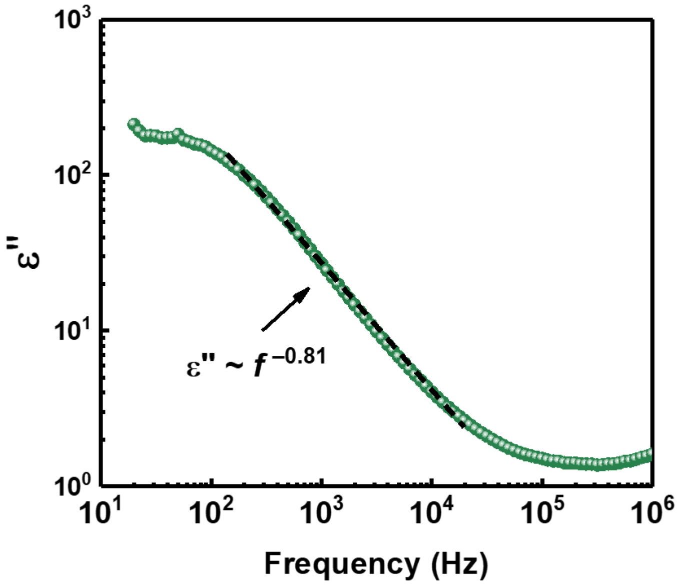

Figure S3. Imaginary part of the dielectric permittivity (ε'') of the untreated PVA (at 373 K). The power-law fit of the high frequency side gives an exponent of 0.81. This number is between 1 and 0.5, which correspond to the extremes of simple drifting (ideal, long-range pathways) and diffusion-limited hopping (tortuous pathways) ion conduction mechanisms, respectively [*J. Chem. Phys.* 124, 144903 (2006); *Macromolecules* 22, 4483 (1989)].

Table S2. Thickness (d) and areal capacitance (C_i) of the gate dielectric upon various treatments.

	Untreated	$SCCO_2$	Annealed	Annealed-$SCCO_2$
d (nm)	342.3	336.0	336.5	325.3
C_i (F cm^{-2})	1.15×10^{-8}	1.02×10^{-8}	1.09×10^{-8}	1.07×10^{-8}

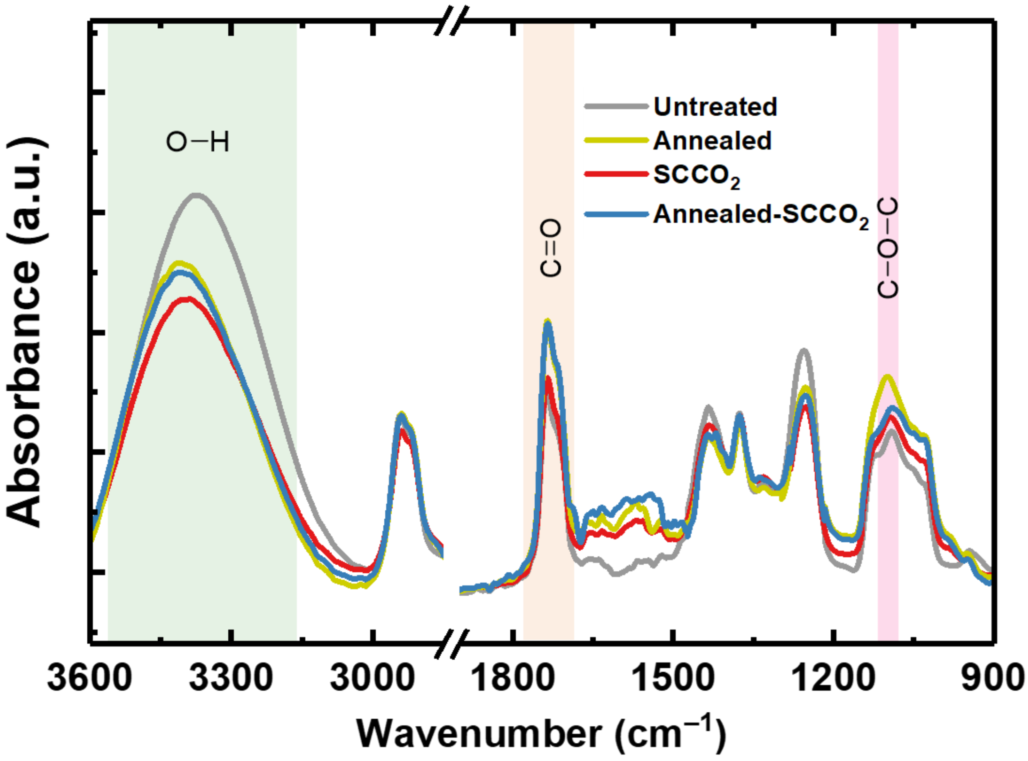

Figure S4. FTIR spectra of the untreated, $SCCO_2$-treated, air-annealed, and sequentially air-annealed and $SCCO_2$-treated PVA films.

Table S3. Summary of the device characteristics for the PVA OFETs fabricated on flexible ITO-coated plastic substrates. The statistics were obtained based on the analysis over 15 devices.

	V_{th} forward (V)	ΔV_{th}	$SS_{forward}$ (V/dec)	I_{on} (nA μm^{-1})	I_{on}/I_{off}	μ_{sat} (cm^2 V^{-1} s^{-1})	μ_{eff} (cm^2 V^{-1} s^{-1})
Untreated	−5.09	5.47±1.77	1.48±0.68	101±18	1.2×10^5	11.4±3.8	7.8±2.6
$SCCO_2$	−1.57	0.03±0.07	0.25±0.11	158±28	9.2×10^5	30.2±4.6	13.8±2.1
Annealed	−3.67	4.80±1.11	1.50±0.45	99±8	1.0×10^6	13.1±3.4	8.1±2.1
Annealed -$SCCO_2$	−2.23	1.62±0.20	0.57±0.26	120±21	1.3×10^6	21.3±1.8	10.0±0.8

Table S4. Comparison of the device performance of the reported flexible OFETs using polymer dielectrics.

Semiconductor	Dielectric	μ_{FE} ($cm^2 V^{-1} s^{-1}$)	SS forward (V/dec)	ΔV_{th} (V)	I_{on}/I_{off}	Reference
Single-crystal C10-DNTT	CYTOP	9	~20	~10	10^5	*Adv. Mater.* 23, 1626 (2011)
TIPS-pentacene	Mylar	0.15	~15		10^3	*Nature Commun.* 3, 1259 (2012)
PDVT-10	PVA	11	~0.4	~0.1	1.2×10^4	*Adv. Mater.* 26, 3631 (2014)
P(NDI2OD-T2)	PS/PVA/PMMA	0.038	~0.5	15.4	2.0×10^4	*ACS Appl. Mater. Interfaces* 7, 10957 (2015)
PQT-12:PEO NFs	PDMS	0.012	~30		1.7×10^3	*Adv. Funct. Mater.* 26, 1445 (2016)
Pentacene	PVPy	0.27	~5		10^4	*Sci. Rep.* 8, 8146 (2018)
DPPT-TT	P(VDF-TrFE)-g-PMMA	1.2	~7	4.4	3.1×10^2	*Adv. Funct. Mater.* 28, 1704780 (2018)
Pentacene	PAA/PI	5.6	0.22		1.4×10^6	*Nature Commun.* 9, 2339 (2018)
C8-BTBT	PVA	7.22	~10	~15	6.5×10^6	*Small*, 14, 1801020 (2018)
Single-crystal C8-BTBT	HPCPS	33.4	~0.3		10^5	*ACS Appl. Mater. Interfaces* 11, 34188 (2019)
**C8-BTBT**	**PVA**	**30.2±4.6**	**0.25±0.11**	**0.03±0.07**	**9.2×10^5**	**This work**

Table S5. Comparison of the device performance of the reported OFETs using the C8-BTBT semiconductor.

Substrate	Semiconductor	Dielectric	μ_{sat} ($cm^2 V^{-1} s^{-1}$)	$SS_{forward}$ (V/dec)	ΔV_{th} (V)	I_{on}/I_{off}	Reference
Si or glass substrate	Single-crystal C8-BTBT	Parylene	31.3	2	0.1	10^7	*Nature* 475, 364 (2011)
	C8-BTBT	Polyimide	0.56	4.6		1.3×10^5	*Phys. Chem. Chem. Phys.* 15, 950 (2013)
	C8-BTBT	PMMA/SiO_2	3.0	~5		10^7	*J. Phys. Chem. C* 117, 12337 (2013)
	C8-BTBT	PVP:HAD	43	~2	~1	10^3	*Nature Commun.* 5, 3005 (2014)
	Single-crystal C8-BTBT	PMMA/SiO_2	52	~9		10^5	*Appl. Phys. Lett.* 106, 193303 (2015)
	C8-BTBT	HPCPS	53	~0.3		10^4	*J. Mater. Chem. C* 7, 4879 (2019)
	C8-BTBT	P(VDF-co-TrFE)	0.032	~5	~3.5	10^5	*Adv. Electron. Mater.* 6, 1901250 (2020)
	**C8-BTBT**	**PVA**	**22.1±2.9**	**0.43±0.21**	**0.00±0.02**	$\mathbf{3.3\times10^4}$	**This work**
Flexible substrate	C8-BTBT	PVA	7.22	~10	~15	6.5×10^6	*Small*, 14, 1801020 (2018)
	Single-crystal C8-BTBT	HPCPS	33.4	~0.3		10^5	*ACS Appl. Mater. Interfaces* 11, 34188 (2019)
	C8-BTBT	CYTOP/PVA	5.83	~2	~3	5.8×10^4	*Adv. Optical Mater.* 8, 1901651 (2020)
	**C8-BTBT**	**PVA**	**30.2±4.6**	**0.25±0.11**	**0.03±0.07**	$\mathbf{9.2\times10^5}$	**This work**

Supplementary Video 1: switching behavior of the OFET-OLED circuit under the square-wave V_G of 16 s in period.

Supplementary Video 2: switching behavior of the OFET-OLED circuit under the square-wave V_G of 0.2 s in period (i.e. 5 Hz square wave).
